# *Streptococcus equi* subsp. *zooepidemicus* finding in confirmed feline infectious peritonitis cat patient

**DOI:** 10.1016/j.heliyon.2021.e07268

**Published:** 2021-06-10

**Authors:** Madarina Wasissa, Fajar Budi Lestari, Siti Isrina Oktavia Salasia

**Affiliations:** a Department of Clinical Pathology, Faculty of Veterinary Medicine, Universitas Gadjah Mada, Yogyakarta, Indonesia; b Department of Bioresources Technology and Veterinary, Vocational College, Universitas Gadjah Mada, Yogyakarta, Indonesia; c Inter-Department of Biomedical Sciences, Faculty of Graduate School, Chulalongkorn University, Bangkok, Thailand

**Keywords:** *Streptococcus equi* subs. *zooepidemicus* (SEZ), Feline infectious peritonitis (FIP), Feline coronavirus (FCoV), Molecular analysis, Manifestation, Diagnosis

## Abstract

**Background:**

Feline infectious peritonitis (FIP) is a fatal immune-mediated disease in cat, caused by mutated feline coronavirus (FCoV). Due to its difficulties in diagnosis, FIP is sometimes underdiagnosed. Therefore, several laboratory procedures were performed to gain high index suspicion of FIP. However, through several laboratory findings, not only FIP but also SEZ infection was confirmed in this case.

**Case description:**

A-year-old male, domestic cat was admitted to Veterinary Medicine Clinical Pathology Laboratory, Universitas Gadjah Mada, for further effusion examination due to its high suspicion of feline infectious peritonitis (FIP). Further examination using molecular and post-mortem analysis resulted on confirmed SEZ infection and FIP. This study informed the manifestation and pathological changes in patient with SEZ and FIP in the same time.

**Conclusions:**

This study showed that viral infection followed by bacterial infection could be fatal and untreatable. After these findings, clinicians may consider SEZ infection in cat with respiratory disorder followed by thoracic effusion besides FIP. Companion animal, especially outdoor-kept animal, possibly become infected from its contact to another human or animal in the environment.

## 1 Introduction

*Streptococcus equi* subsp. *zooepidemicus* is known as an opportunistic pathogen that cause fatal purulent disease in wide variety mammals including horses, pigs, monkey, llama, sheep, goats, and human (Barnham et al., 1987; Hewson and Cebra, 2001; Pelkonen et al., 2013; Rasmussen et al., 2013; Salasia et al., 2004; Soedarmanto et al., 1996). It has been also associated with food poisoning disease which contaminates dairy products causing septicemia and arthritis in humans (Kuusi et al., 2006). Additionally, SEZ which belongs to Lancefield Group C streptococci infection is reported in companion animal. An outbreak in the kennel due to SEZ was reported causing acute hemorrhagic and purulent pneumonia (Kim et al., 2007). The evidence showed its transmission from dog to human was also reported (Abbott et al., 2010). SEZ infection in cats was previously rarely investigated until several outbreaks in catteries occurred (Blum et al., 2010; Britton and Davies, 2010). According to the previous reports, SEZ infection in cats caused suppurative pneumonia, rhinitis, and meningitis (Frymus et al., 2015). Several reports suggested β-lactam antibiotics to treat streptococcal infection and trimethoprim-sulfamethoxazole for infection that involve the brain; nevertheless, sensitivity test should be performed to give effective therapy (Frymus et al., 2015; Martin-Vaquero et al., 2011).

Feline infectious peritonitis (FIP) is a fatal immune-mediated disease caused by virulent feline coronavirus (FCoV) or well-recognized as FIP virus (FIPV), characterized by granulomatous lesion in several organs and vasculitis accompanied with or without effusion in body cavities (Kipar and Meli, 2014). FCoV, a highly-mutated single-stranded RNA virus, has been known with two serotypes existed which type 1 FCoV is the most prevalent worldwide (Addie et al., 2003). Serotype 2 is a combination between type 1 FCoV and CCoV (Herrewegh et al., 1998). The serotype is distinguished by its spike (S) gene and protein as the binding site between virus and cell host. Due to its mutation, FCoV has ability to bind to monocytes or macrophages and spread systematically that subsequently induce complex immune response. The involvement of a complex immune responses resulted in various unspecific clinical signs. In addition, FIP virus may induce immunosuppression that leads to opportunistic pathogen complicating the diagnosis or worsening the disease (Pedersen, 2009).

FIP is one of the most challenging diseases in cat due to its difficulty in either diagnosis or treatment. Due to its unspecific clinical signs including lethargy, inappetence, recurrent fever, and progressive weight loss, antemortem diagnosis FIP needs confirmations from several procedures to gain a high index of suspicion of FIP. Several methods to detect FCoV infection has been reported previously including molecular detection using RT-PCR targeting specific gene such as 3-UTR, S gene, M gene, or non-structural protein (Herrewegh et al., 1995; Addie et al., 2003; Simons et al., 2005; Maia et al., 2013; Tanaka et al., 2015) and immune-staining as the gold standard method (Kipar et al., 1998; Sharif et al., 2010). Most treatment of FIP is supportive which temporarily reducing clinical signs and immune-modulator to stimulate diseased patient's immunity (Pedersen, 2014). The recent investigation that gives a positive result in survival rates and clinical cures is antiviral broad-spectrum coronavirus protease inhibitor GC376 and the adenosine nucleoside analogue GS-441524 (Izes et al., 2020).

According to the previous reports, bacterial infection is secondary etiological agent in respiratory disease in cat (Greene, 2012). Therefore, in some situations, to obtain a definitive diagnosis and the best knowledge about the agent, further laboratory investigation is needed. This case report highlights the evidence of FIP with SEZ as its concomitant infection through clinical signs, laboratory findings, and pathological changes causing fatal manifestation in cat. The demonstrated result supports the clinicians to consider SEZ infection in cat with the similar clinical manifestation. In addition, this occurrence underlines the SEZ infection in companion animal that is potentially zoonosis yet so far considered unimportant in Indonesia. Therefore this case encourages the next further investigation of SEZ infection in companion animal in Indonesia.

## 2 Case presentation

### 2.1 Patient and clinical signs

A-year-old, male, domestic cat was admitted to Veterinary Medicine Clinical Pathology Laboratory, Universitas Gadjah Mada, for further examination of abdominal and pleural effusion due to its high suspicion of feline infectious peritonitis (FIP) along with its consistent history, clinical signs, and physical examination. The cat was kept outdoor with no limited access to the surrounding environment. According to the owner's information, the cat behaved oddly for the last two months and followed with lethargy, progressive weight loss, respiratory disorder, and abdominal distension. The disease investigation was part of FIP research project that all procedures were under Faculty of Veterinary Medicine Universitas Gadjah Mada Ethical Committee supervision (005/EC-FKH/06/2017). The data collected related to the patient and its publication was under the owner's consent.

### 2.2 Laboratory findings

To gain a definitive diagnosis, abdominal and thoracic effusions were aseptically collected for physical examination, Rivalta's test, cytology analysis, microbial culture, and molecular detection. Due to its severe condition, the moribund cat was died after examined. A post-mortem examination was respectively done under the owner's consent.

Both thorax and abdominal effusions were exudate-type positive using Rivalta's test. Cytology analysis of thorax effusion showed cloudy exudate which contained macrophages, mesothelial cells, degenerated neutrophils, and intracellular and extracellular round cell bacteria. The abdominal fluid was tan-colored, sticky exudate, consisting mostly of macrophages, mesothelial cells, and degenerated neutrophils with eosinophilic proteinaceous background.

Microbial culture resulted positive for thorax effusion but remained negative for abdominal effusion. According to the cytology of thorax effusion analysis, bacterial culture isolation was focused on streptococcal or staphylococcal bacteria due to the only round shape, intracellular, and extracellular bacterial found. For further bacteria species confirmation, bacterial DNA was extracted from pure bacterial isolate. PCR analysis, targeting *Streptococcus equi* subs. *zooepidemicus* in 560 bp V2 fragment 16 rRNA was performed. The confirmed SEZ was cultured in Mueller Hinton Agar (MHA) for antimicrobial susceptibility test with disk diffusion. The result indicated that the isolate was still susceptible to gentamycin, tetracycline, erythromycin, and cefoxitin, yet resistant to ampicillin, penicillin-G, and oxacillin. The resistance interpretation was obtained following the manufactured antimicrobial susceptibility disk information sheet (Oxoid) and performance standard for antimicrobial susceptibility testing (CLSI, 2017) which showed no inhibition zone for ampicillin (10 μg), penicillin-G (10 U), and oxacillin (5 μg). However, the inhibition zone was greater than 15 mm for gentamycin (10 μg), tetracycline (30 μg), erythromycin (15 μg), and cefoxitin which could be considered as susceptible.

Definitive diagnosis of FIP was based on consistent history, clinical signs, molecular detection of FCoV, post-mortem findings, and histopathology using immunohistochemistry analysis. Molecular analysis was performed using primer listed in Table 1 resulted in confirmed FCoV type 2 which is considered a rare case due to mostly FCoV infection in Indonesia is FCoV type 1 (unpublished data).

**Table 1:** The primer that were used in this investigations.

Target	Primer	Nucleotide	Size	Reference
FCoV 3 ‘UTR	P205	GGCAACCCGATGTTTAAAACTGG	223 bp	Herrewegh et al. (1995)
	P211	CACTAGATCCAGACGACGTTAGCTC		
	P276	CCGAGGAATTACTGGTCATCGCG	177 bp	
	P204	GCTCTTCCATTGTTGGCTCGTC		
FCoV S gene	Iffs	GTTTCAACCTAGAAAGCCTCAGAT	Type 1 376 bp Type II 283 bp	Addie et al. (2003)
	Icfs	GCCTAGTATTATACCTGACTA		
	Iubs	CCACACATACCAAGGCC		
	nIffles	CCTAGAAAGCCTCAGATGAGTG	Type 1 360 bp Type II 218 bp	
	nIcfs	CAGACCAAACTGGACTGTAC		
	nIubs	CCAAGGCCATTTTACATA		
16 rRNA SEZ	V2-f	GAGAGTTTGATCCTGGCTCAGCA	560 bp	Blum et al. (2010)
	V2-r	TTACCGCGCGGCTGCTGGCACGT		

**Figure 1:**
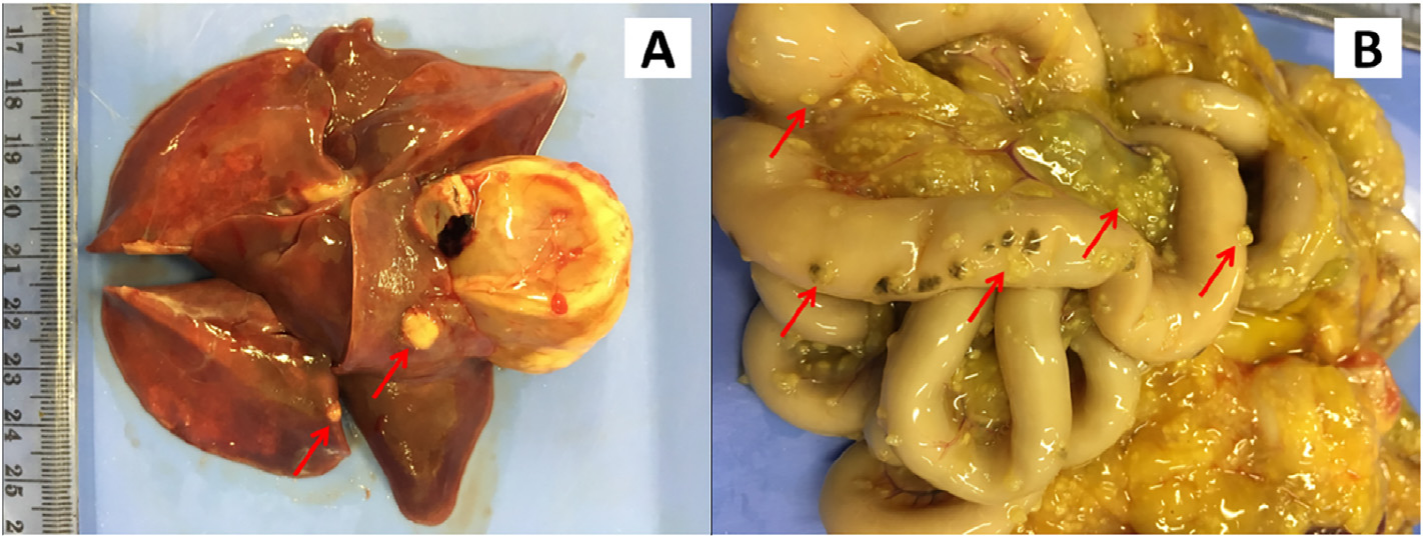
Gross examination of the patient showed pyogranulomatous nodule (red arrows) were diffusely found in lungs (A) intestine and mesentery (B).

**Figure 2:**
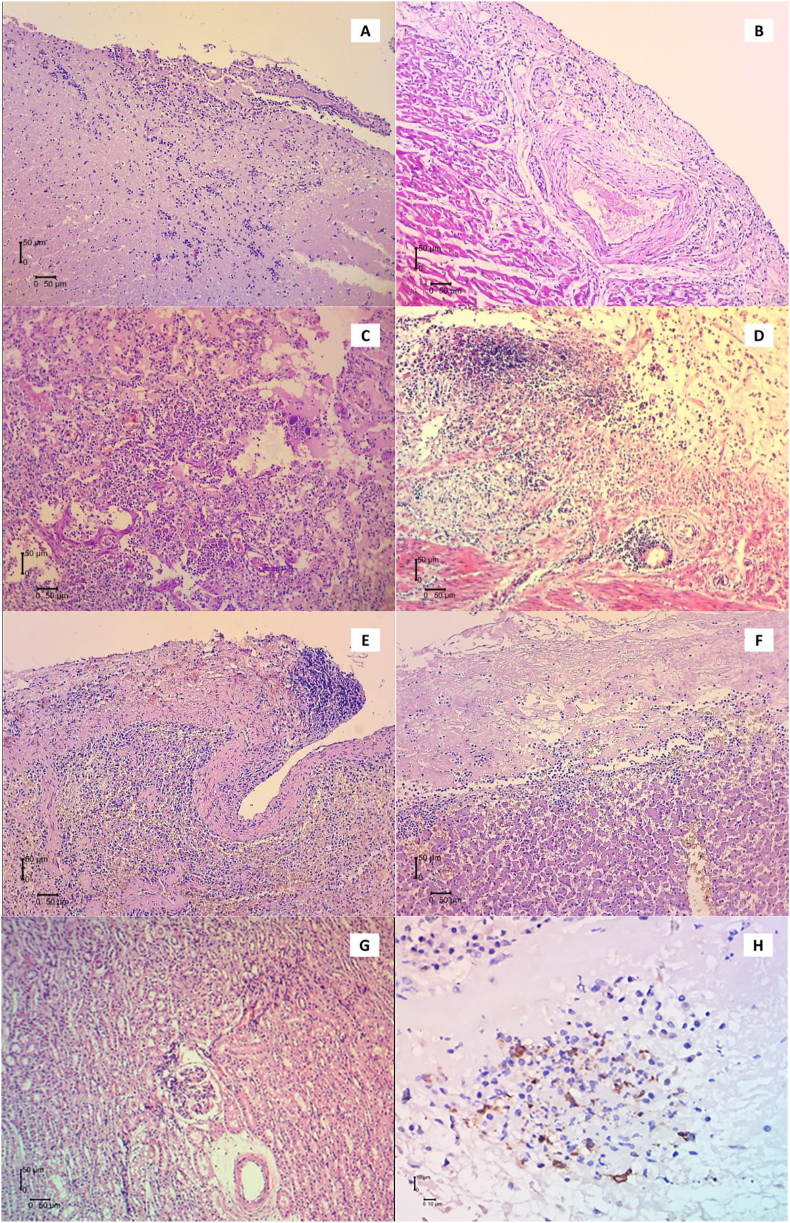
Histological findings from several organs with H&E staining (A, B, C, D, E, F, and G) and immunohistochemistry for FCoV staining (H). A Infiltration of lymphocytes in meninges. B Thickening of heart capsule with inflammatory cells. C Suppurative broncho-pneumonia with degenerated neutrophil, necrotic debris inside alveolar septa. D Pyogranulomatous vasculitis was found in intestinal serosa containing necrotic area surrounded by inflammatory cells. E Thickening spleen capsule with pyogranulomatous lesion. F Thickening liver capsule with pyogranulomatous lesion and vasculitis in hepatic vein. G Mild inflammation in kidney parenchymal part. H Immunopositive with FCoV contained macrophages were detected in necrotic area of liver capsule.

Post-mortem examination showed grossly damage in internal organs consistent with FIP (Figure 1) including the lungs. Tissue samples from lung, spleen, intestine, liver, and kidneys were taken, processed, and stained with H&E and immunohistochemistry for FCoV special stain. Histologically, the organs examined were markedly thickening in the capsule with inflammation cells and necrotic areas (Figure 2B, 2D, 2E, 2F). Suppurative broncho-pneumonia in lungs was marked with exudate charge containing degenerated neutrophils, necrotic debris, and abundant macrophages inside the bronchi and alveolar septa (Figure 2C).

Pyogranulomatous vasculitis was found in intestinal serosa and intestinal mesenteries consisted of necrotic areas surrounded by inflammatory cells. Granulomatous disease progress presented in the spleen as well as in other organs with thickening of its capsule, necrotic lesions, and accumulation of monocytes, lymphocytes, and neutrophils. Vascular changes were also seen in the liver and kidneys tissue where the inflammatory cells surrounded the veins.

Histology using immune-staining was performed using the previously mentioned method (Kipar et al., 1998) to confirm FIP virus infection. Immunohistochemistry was conducted using micro-polymer HRP/DAB detection kit (ab236466, Abcam, UK) and monoclonal mouse anti-coronavirus (FIPV3-70. 1:100, Invitrogen, CA, USA) to detect FCoV in the tissue sample. Figure 2H demonstrated a positive result marked by darker color of viral-contained macrophages and precipitates within granuloma in the liver capsule.

## 3 Discussion

Feline infectious peritonitis (FIP) is a fatal immune-mediated disease caused by mutated feline coronavirus (FCoV) which is characterized by peritonitis, fibrinous effusion in the body cavity, and pyogranulomatous lesion in several organs (Kipar and Meli, 2014). Ante-mortem diagnosis of FIP is extremely frustrating, especially for clinicians with limited access to advance laboratory facilities or limited to owner financial problems. Due to its difficulties in diagnosis, FIP is rarely reported and sometimes underdiagnosed in Indonesia. Until today, postmortem histopathology is still considered as the gold standard to obtain a definitive diagnosis. However, the method is not practical and is considered too invasive.

Due to its problematic diagnosis, excluding the differential diagnosis of FIP is more preferable to gain high index suspicion of FIP rather than using an invasive method such as abdominal organ biopsies to obtain a definitive diagnosis. The analysis of history, clinical signs, and laboratory findings could help clinicians to decide further examination (Tasker, 2018). In this study, bacterial culture was performed to exclude bacterial peritonitis or bacterial pneumonia. However, according to the findings, bacterial pneumonia cannot exclude FIP virus infection.

In this investigation, molecular detection of FCoV was performed using nested RT-PCR targeting highly conserved 3′ untranslated region (3′UTR) of the end of the FCoV genome (Herrewegh et al., 1995). Molecular detection cannot be the solely tool to obtain definitive diagnosis of FIP however, it could be helpful support to get more evidence. Additionally, FCoV type 2 was detected in this case. According to previous study, type 2 FCoV is a recombination of type 1 FCoV and CCoV (Herrewegh et al., 1998). Further investigation is needed to obtain better knowledge about the viral type that circulates and its relation to the infection in Indonesia.

In the histopathology findings, brain tissue did not show strong immunolabeling for FIPV. However, it cannot be concluded that the brain was FIPV infection-free. In the earlier reports mentioned that FIPV was responsible for several neurological damages marked by perivascular inflammation, meningitis, encephalitis, leptomeningitis, or effusive accumulation (Crawford et al., 2017; Diaz and Poma, 2009; Foley et al., 2003; Mesquita et al., 2016; Rissi, 2018; Timmann et al., 2008). The absence of viral antigen in the brain was probably caused by randomly distributed viral antigen within tissue lesion. Whereas SEZ infection was reported causing leptomeningitis with mixed inflammation cells found in brain tissue (Britton and Davies, 2010). Thus, in this case, the lesion was presumably compounded by SEZ infection.

Based on the findings, it was difficult to differentiate the manifestation was individually caused by SEZ or FIP alone in a certain organ. It was presumed that SEZ was the secondary infection of FIP due to bacterial infection is commonly a secondary infection in respiratory disease (Greene, 2012). Nevertheless, this finding would be helpful to clinician to consider SEZ infection in respiratory disease in cat. It may help for antimicrobial choices knowing SEZ is still susceptible to several antibiotics.

Further investigation about SEZ is related to the public health consideration. According to the owner's information, the cat usually wandered around the surrounding environment without limited contact with humans or animals. The environment was an urban area with several stray cats around the neighborhood but there was no other cat showing clinically ill at the same time. Previously evidence, SEZ transmission is interspecies such as horse to human (Rose et al., 1980), human to animal (Barnham et al., 1987, 1989), or dog to human (Abbott et al., 2010). In this case, SEZ infection was presumably transmitted from animal, human, or contaminated food from its surrounding environment.

## 4 Conclusion

FIP is still a challenging disease to overcome due to its difficulties in diagnosis and treatment. The viral infection that interferes with the host's immune system encourages the opportunistic pathogen to complicate the disease either in diagnosis or treatment. The findings suggest the clinician to consider SEZ infection in cat with respiratory disorder followed by thoracic effusion besides FIP. SEZ infection is possibly due to its contact with another human or animal in the environment. Therefore, further investigation is needed regarding its importance of public health consideration.

## Declarations

### Author contribution statement

All authors listed have significantly contributed to the investigation, development and writing of this article.

### Funding statement

Madarina Wasissa was supported by PMDSU Scholarship No. 2953/UN1.DITLIT/DIT-LIT/LT/2019 from the Ministry of Education and Culture of the Indonesian Government.

Fajar Budi Lestari was supported by BPP-LN Scholarship from the Ministry of Education and Culture of the Indonesian Government.

### Data availability statement

Data will be made available on request.

### Declaration of interests statement

The authors declare no conflict of interest.

### Additional information

No additional information is available for this paper.
